# Evaluation of the effect of patient education on rates of falls in older hospital patients: Description of a randomised controlled trial

**DOI:** 10.1186/1471-2318-9-14

**Published:** 2009-04-24

**Authors:** Anne-Marie Hill, Keith Hill, Sandra Brauer, David Oliver, Tammy Hoffmann, Christopher Beer, Steven McPhail, Terry P Haines

**Affiliations:** 1 School of Health and Rehabilitation Sciences, The University of Queensland, Brisbane QLD 4072, Australia; 2 School of Primary Health Care, Monash University, Victoria 3800, Australia; 3 Southern Health, Monash University, Victoria 3168, Australia; 4 Princess Alexandra Hospital, Queensland Health, GPO Box 48, Brisbane, Queensland 4001, Australia; 5 School of Physiotherapy, La Trobe University and Northern Health, Bundoora, Victoria 3086, Australia; 6 Preventive and Public Health Division, National Ageing Research Institute Australia, PO Box 2127, Royal Melbourne Hospital, Victoria 3050, Australia; 7 Institute of Health Sciences, City University, London, E1 2EA, UK; 8 Faculty of Medicine, Dentistry and Health Sciences, University of Western Australia, 35 Stirling Highway, Crawley, WA 6009 Perth, Australia

## Abstract

**Background:**

Accidental falls by older patients in hospital are one of the most commonly reported adverse events. Falls after discharge are also common. These falls have enormous physical, psychological and social consequences for older patients, including serious physical injury and reduced quality of life, and are also a source of substantial cost to health systems worldwide. There have been a limited number of randomised controlled trials, mainly using multifactorial interventions, aiming to prevent older people falling whilst inpatients. Trials to date have produced conflicting results and recent meta-analyses highlight that there is still insufficient evidence to clearly identify which interventions may reduce the rate of falls, and falls related injuries, in this population.

**Methods and design:**

A prospective randomised controlled trial (n = 1206) is being conducted at two hospitals in Australia. Patients are eligible to be included in the trial if they are over 60 years of age and they, or their family or guardian, give written consent. Participants are randomised into three groups. The control group continues to receive usual care. Both intervention groups receive a specifically designed patient education intervention on minimising falls in addition to usual care. The education is delivered by Digital Video Disc (DVD) and written workbook and aims to promote falls prevention activities by participants. One of the intervention groups also receives follow up education training visits by a health professional. Blinded assessors conduct baseline and discharge assessments and follow up participants for 6 months after discharge. The primary outcome measure is falls by participants in hospital. Secondary outcome measures include falls at home after discharge, knowledge of falls prevention strategies and motivation to engage in falls prevention activities after discharge. All analyses will be based on intention to treat principle.

**Discussion:**

This trial will examine the effect of a single intervention (specifically designed patient education) on rates of falls in older patients in hospital and after discharge. The results will provide robust recommendations for clinicians and researchers about the role of patient education in this population. The study has the potential to identify a new intervention that may reduce rates of falls in older hospital patients and could be readily duplicated and applied in a wide range of clinical settings.

**Trial Registration:**

ACTRN12608000015347

## Background

Accidental falls are one of the most frequent adverse events reported in hospitals[1] with falls rates ranging from 2.2 falls per 1000 patient days on general acute medical wards[2,3] and up to 20 falls per 1,000 patient days on rehabilitation wards being reported.[4,5] Up to 30% of hospital falls result in injury[6] and fractures sustained from falls in hospitals have recently been found to result in poorer outcomes than fractures sustained from falls in the community.[7] In addition, falls are associated with poorer rehabilitation outcomes, increased length of stay (LOS) in hospital, increased costs, litigation, and increased risk of institutionalization.[1,8,9] Falls in the immediate period after discharge are also common for older people, further delaying recovery from hospitalisation,[10,11] with frequent injury and high levels of health care resource use also recorded.[12]

Recent meta-analyses have identified that there is only limited, if any, evidence for reduction of falls amongst hospital patients.[13,14] The small numbers of multifactorial randomised controlled trials (RCTs) conducted to date have included some large well designed studies that have demonstrated conflicting results. [15-18] These studies have used a variety of multifactorial interventions including, but not limited to, risk factor assessment followed by interventions targeted at each identified risk factor, patient exercise, education and environmental modification resulting in difficulty identifying the effective components. There is yet to be a large trial to determine the effect of a single intervention in this setting.

Falls prevention for older people following hospitalisation has been assessed in a limited number of randomised trials with equivocal results. [19-23] Patients followed up after discharge by an occupational therapist, who provided home environment modifications and training, showed a reduction in rates of falls,[20,21] but in contrast, a similar randomised trial only showed an effect in a subgroup of recurrent fallers.[23] In-hospital preparation is a feasible intervention for reducing falls in older patients after discharge. However a multifactorial programme, including systematic assessment and treatment of falls risk factors,[19] and a comprehensive geriatric assessment with follow up treatment[22] failed to reduce post discharge rates of falls.

Patient behaviour is an integral contributing factor to many in-hospital falls.[24,25] Thus strategies to modify patient behaviour may be an effective means to reduce rates of falls. Patient education is frequently cited as one component of multifactorial interventions.[15,17,26] Subgroup analysis of a previous randomised trial of a multifactorial falls prevention programme suggested that patient education was the most effective component in reducing falls.[27] However there has rarely been description of the theoretical frameworks underpinning development of content, and approaches to the delivery of education in trials to date. Thus there are few insights into the mechanisms by which it is thought patient education programs may reduce the rate of falls. Presenting this information would allow more reliable differentiation between successful and unsuccessful elements of content, and approaches to the provision of education programmes.

This study is the first randomised controlled trial to evaluate the effect of individual patient education for the prevention of falls in the hospital setting and also to investigate its effect on falls after discharge. The study will examine the optimal provision of patient education in this setting. It will also examine a theoretical framework that explains preventative health behaviours in order to understand falls after discharge.

### Research Questions

#### Primary research question

1. Does providing individual patient education for older patients in addition to usual care affect the rate of falls while in hospital compared to providing usual care alone?

#### Secondary research questions

2. Does providing individual patient education for older patients in addition to usual care affect the rate of falls after discharge compared to providing usual care alone?

3. Does providing individual patient education in addition to usual care affect patient length of stay in hospital or change in health related quality of life during admission?

4. Does providing individual patient education for older patients in addition to usual care influence patients' pre – discharge self – perceived risk of falls, knowledge about falls and falls prevention, motivation to engage in self protective strategies in the first six months after discharge, or their levels of participation in falls prevention activities after discharge?

5. Does providing face-to-face health professional follow up in addition to multimedia patient education and usual care reduce falls compared to providing multimedia patient education and usual care?

## Methods and design

### Study Design

**Figure 1:**
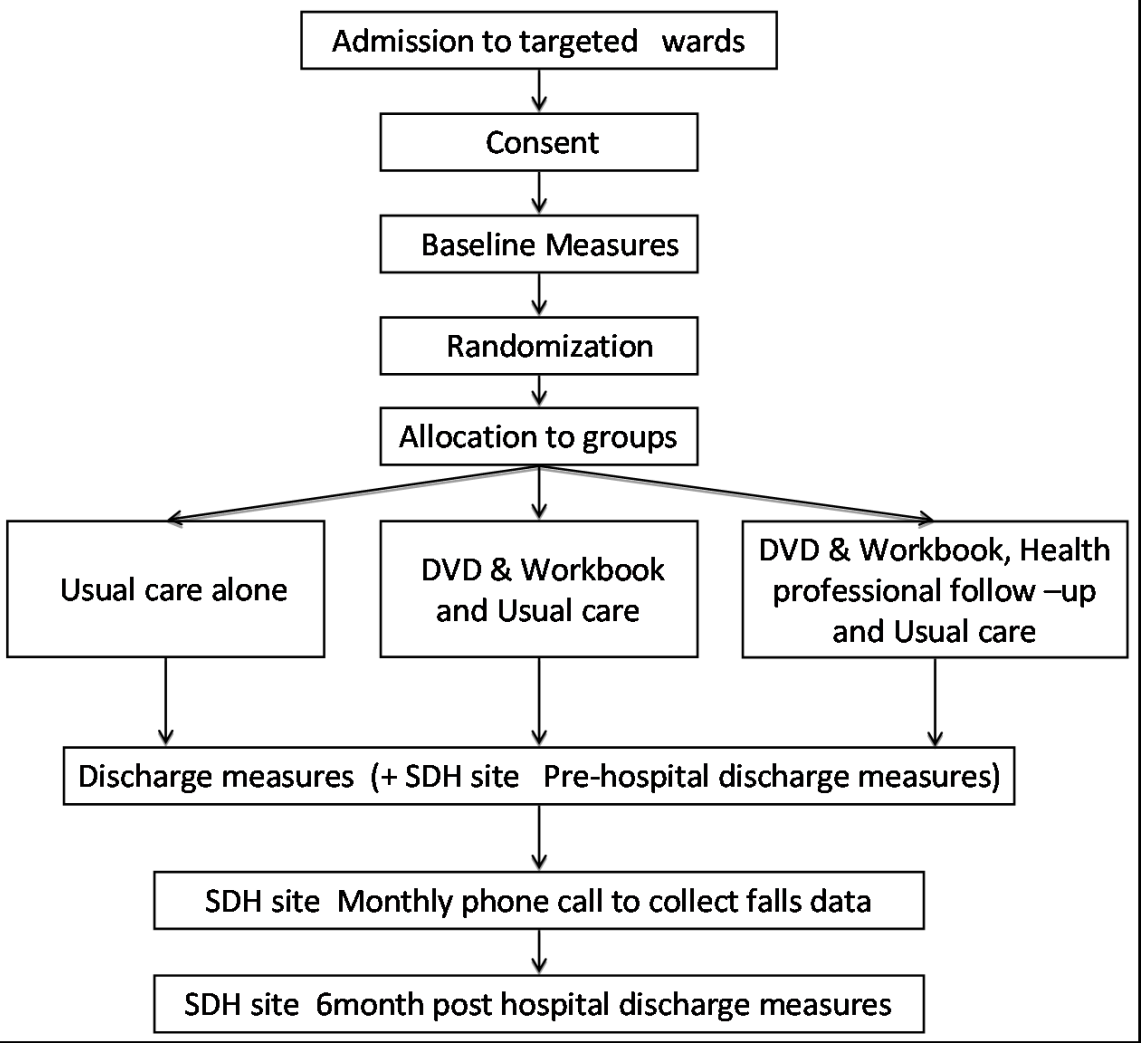
**Participant flow through the study**.

This study is a three group prospective randomised controlled trial (see Figure 1), with blinded weekly follow-up and discharge assessments of participants in the hospital setting. In a sub-trial, participants at one site are subsequently followed up at home monthly by blinded assessors over a 6 month period.

### Location and Setting

Patients are being recruited from the geriatric assessment and rehabilitation unit, orthopaedic unit and acute/respiratory medicine unit of Princess Alexandra Hospital (PAH), Brisbane, Queensland and the rehabilitation and stroke unit, and medical and surgical wards of Swan Districts Hospital (SDH), Perth, Western Australia.

PAH is a 700 bed tertiary facility in Queensland and SDH is a 194 bed general hospital, serving the eastern region of the Perth metropolitan area.

The acute wards at both hospitals admit older patients who are undergoing short stay surgical and medical treatment for a variety of cardiac, respiratory and orthopaedic conditions or other medical or surgical diagnoses. The sub acute rehabilitation wards and stroke units admit older patients who are undergoing acute care for stroke, or longer rehabilitation for a variety of geriatric conditions including cardio-respiratory conditions, fractures, falls, stroke, Parkinson's disease and other geriatric management.

### Population and Recruitment

Patients eligible for inclusion in the study are individuals who are admitted to a participating ward at PAH or SDH, are 60 years of age or older, are able to provide written consent (or, for patients with cognitive impairment, have a family member/carer who can provide written consent) and have not previously been enrolled in the study. Recruitment commenced in January 2008 and there are currently 1150 participants enrolled in the trial.

### Randomisation

Participants are allocated to groups in a 1:1:1 ratio in consecutive order after enrolment by the research assistant. The allocation is performed by the site investigator (AMH and SM) opening an opaque sealed envelope with the participant's study identification number on it. The paper inside the envelope contains the group allocation. The envelope order is determined by a computer generated random number sequence that was produced by the senior investigator (THa) who is not involved in recruitment, intervention or data collection.

The research assistants, who enrol patients, collect inpatient falls data and conduct pre- discharge assessments and surveys, are blinded to the group allocation throughout the study including the post discharge data collection and final survey at the 6 month period. The site investigators are not involved in data collection.

### Intervention/Control conditions

#### 1. Usual care

Usual care on the medical and surgical wards consists of 24 hour availability of nursing and medical care, daily physiotherapy, and referrals to other allied health professionals as required. On the restorative and stroke units there are also daily occupational therapy sessions, and social work assessment and management provided. Therapy on all wards is usually provided 5 days per week. Locally developed falls risk assessment tools (unpublished) are used at both sites and special arm bands are provided to patients assessed as being at high risk for falls.

#### 2. Interventions

##### • Patient education via Digital Video Disc (DVD) and written workbook

Patient education is provided via a DVD and a written workbook. The education materials are based upon prior work of THa and KH,[6,27] but were further developed and refined by THa with contribution from other team members. The DVD and the workbook were designed to contain identical and complementing content. These materials have been tested for clarity, ease of use, and impact on patient knowledge, self-perceived risk of falls and motivation to prevent falls in a previous randomised trial (n = 100) conducted by the investigative team.[28] This study found that the use of the video-based materials led to superior outcomes than use of the written materials alone. Evaluation that accompanied our previous study revealed that 98% of participants who viewed the DVD strongly agreed or agreed that the DVD narration used words they could understand and 95% strongly agreed or agreed that the DVD footage made it easier to understand the narration. All participants who viewed the workbook strongly agreed or agreed that the book used words they could understand and 98% strongly agreed or agreed that the book was well set out.

**Figure 2:**
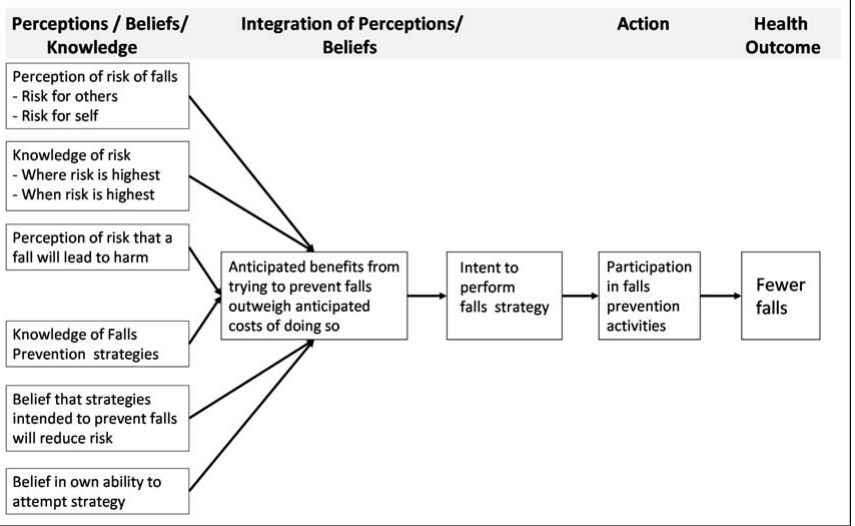
**Health Belief Model adapted to falls prevention education**. The constructs of the HBM applied to the application of the education intervention.

The content included in the education was designed using the constructs of the Health Belief Model (HBM)[29] aiming to facilitate behaviour change among participants who use the materials. The HBM is a widely used framework for predicting preventative health behaviours [29,30] and for developing interventions to facilitate behaviour change.[31] A conceptualisation of the HBM as applied to promoting falls prevention activities amongst hospital patients is presented (see Figure 2).

Specifically, the content:

a) Informs participants of the risk of falls and fall-related harm to provide participants with accurate information regarding the risks they face,

b) Informs participants of falls prevention strategies that they could undertake within the hospital setting,

c) Fosters participant belief that they could successfully undertake falls prevention strategies and that if undertaken, their risk of falling will reduce,

d) Provides a cue for action by facilitating participant planning to undertake falls prevention strategies.

Information presented under aims a) and b) is based upon local data and data presented in previous research.[7,24,25]

The workbook follows the recommended principles of design for written patient education materials and can be used by people with low functional health literacy.[32,33] A 20 point font in Arial and 1.5 line spacing is used within an A4 format (24 pages in total). The workbook contains a mixture of text, colour graphics and photographic images printed on matt paper. The readability level of the workbook was assessed at 7^th ^grade level using the SMOG readability formula.[34]

In the DVD, words are spoken verbatim and the manner of speech follows recommended principles for clear oral communication.[33] The DVD is 14 minutes in duration and was edited using Pinnacle Studio Plus version 9 software. The DVD is played on a portable Digital Video Disk Player (Dick Smith Electronics, Australia, Model: DSE 9" G7137) that is loaned to the patient. Accompanying stereo headphones (Sony Australia, Model: MDRXD 100) minimise interference of ambient sounds from the hospital ward environment and transfer of information to other participants who may be in the control group. Headphones also assist delivery of the education to participants with sensory impairments.

Video presentation of information caters to patients with different learning styles (auditory, visual) and assists patients who have low functional health literacy, English as a second language or visual impairment to understand the content. The optimal way to retain information appears to be to hear it, see it, have it repeated and interact with it.[35,36]

##### • Health professional follow up visits

After the patient education is provided using the DVD and workbook, participants in group 3 receive follow up sessions conducted by a physiotherapist who has experience in hospital care for older patients and received training from the principal investigator prior to the study commencement. The physiotherapists have discretion over the duration of each participant session (usually between 15 and 35 minutes), and the total number of sessions provided (though the content of the program is intended to be covered in 4 sessions). Duration and number of training sessions may be influenced by personal, medical and environmental factors relevant to each participant. The progression of each session is based on the theoretical frameworks of the HBM specifically:

a) Perception of risk – which facilitates participants having an accurate perception of the risk of falls in hospital and appropriately identify their personal susceptibility to falls and fall-injury.

b) Knowledge of the risk and of the self protective strategies required to reduce the risk – which facilitates knowledge of the nature and mechanisms of falls and of current known falls prevention strategies.

c) Belief that using strategies will reduce risk – which facilitates the participant translating their knowledge into practical achievable short and longer term goals to prevent falls and promote safety in the hospital.

d) Facilitation of confidence and motivation to take action to reduce risk – which empowers and supports participants to act on their planned strategies.

### Outcome measures

The primary outcome measure is participant falls during both hospitalisation and the 6 month post discharge period. The definition of a fall used in this study is the World Health Organisation definition namely: "any event when the participant unexpectedly comes to rest on the ground, floor or another lower level."[37]

Secondary outcome measures being collected include length of stay in hospital, and change in health-related quality of life during hospitalisation, quantified using the EQ-5D instrument.[38] The EQ-5D is administered by face-to-face interview, with the question set presented to the participant in hard copy and read out by a research assistant.

Secondary outcome measures for participants in the home follow up sub-trial at the SDH site include a pre-discharge assessment using a survey (see additional file 1) of their self perceived risk of falls and falls injuries, knowledge about falls mechanisms, awareness of strategies to prevent falls at home and confidence and motivation to prevent falls at home after discharge. The survey items were developed by the investigative team during a previous randomised trial of the education materials.[28]

The final outcome survey (see additional file 2) administered to participants in the home follow up trial measures participants' engagement in falls prevention activities. The activities specified in the survey items were selected on the basis of current evidence for falls risk reduction when implemented amongst older community dwelling people.[39,40]

Demographic data being collected includes age, gender, falls in the 6 months prior to hospitalisation, admission and discharge residential living status, cognitive status using the Short Portable Mental Status Questionnaire,[41] mood using the Geriatric Depression Scale,[42] and medical diagnosis on admission.

### Procedure

Prior to commencement of recruitment, hospital staff working in participating wards were trained to apply the WHO definition of a fall, using 14 video-scenarios. This training was necessary as previous research indicates that absence of a definition is a barrier to documenting falls and that providing a written definition of a fall in itself does not considerably improve agreement between hospital staff as to what constitutes a fall.[43,44]

Baseline measurements are collected from participants after written consent has been obtained (or in the case of patients with cognitive impairment, where their carer or family has provided written consent). Participants are then randomised into one of the 3 groups. Participants in group 1 then continue to receive 'usual care'. Participants in group 2 receive the patient education via DVD and written workbook in addition to usual care. These participants have an opportunity to view the DVD within 24 hours of being allocated to this intervention. If the participant is too unwell to view the DVD immediately, they are provided with an opportunity to view the DVD as soon as practical after their health condition stabilizes. Participant family members, care-givers, or guardians are also invited to view the DVD and the workbook on regular workdays. Participants are asked if they wish to view the DVD again on a weekly basis until discharge and are encouraged to review their goals via interactive use of their workbook. The intervention is identical to that provided for participants in group 3 with the exception that there is no health professional follow-up visit. Participants in group 3 receive the patient education intervention described in group 2 and also receive the individual health professional follow-up visits. The health professional monitors these visits for time, content and participants' knowledge and achievement of their planned strategies.

This is a pragmatic trial and the interventions are provided in the usual hospital environment, most often at the bedside. Participants receive the intervention at a convenient time for optimal engagement. For example, if a participant becomes acutely unwell the investigator returns to complete the intervention when the participant is medically stable.

Research assistants, who are blinded to group allocation, check for falls occurrence on a weekly basis by reviewing medical records and interviewing staff and participants and their families. Periodic review of computerised hospital incident reporting systems is also undertaken.

At point of discharge, participants at the SDH site are administered the pre-discharge survey, given a falls diary and trained in how to record information in the diary. Participants and their families are subsequently followed up monthly by telephone for 6 months after discharge by research assistants to check for falls after discharge. Research assistants receive training from the investigator to question participants regarding falls occurrence and the circumstances of each fall reported. Participants in the home follow up trial are also administered the final outcome survey via telephone at 6 months post discharge from hospital. If participants have cognitive impairment, their carers assist them to respond to survey items. This procedure aims to maximise accuracy of the data collected about the participants' engagement in falls prevention activities.

A cross-sectional survey of hospital nursing, physiotherapy, occupational therapy, and medical staff will be conducted at the conclusion of subject recruitment to gauge level of staff awareness of participant group allocation. The survey will be conducted once only and hospital staff are informed of this so that they are not tempted to actively identify patient group allocation in order to respond more accurately in assessments they may anticipate taking place in future.

### Data Analysis

#### Primary Outcome

Data analysis will be performed on an intention-to-treat basis using a two-tailed alpha = 0.05. Falls rates in hospital will be analysed using Cox semi-parametric proportional hazards regression analysis (Anderson-Gill model for recurrent events) with the intervention groups entered as dummy variables. This allows multiple falls by individual participants, variations in patient length of stay, and time varying covariates to be incorporated into the analysis if required. Nelson-Aalen cumulative hazard plots will also be developed to graphically display cumulative fall rates over time for each of the groups. Visual analysis of these plots and the Schoenfeld residuals test will be used to check the proportional hazards assumption with this data. The proportion of participants having one or more falls in each group (being a faller) will be compared between groups using relative risk (95% confidence intervals).

#### Secondary Outcomes

Post discharge falls data from participants recruited at the SDH site will be analysed using the same approach as falls data collected while participants are in hospital.

Length of stay in hospital will be compared between groups using Cox semi-parametric regression analysis (single event) which permits adjustment for covariates as required. Health-related quality-of-life assessments undertaken using the EQ-5D will be converted into summative "utility" scores using the Dolan formula.[45] Change in health-related quality-of-life during hospitalisation will be compared between groups using linear regression where the discharge measure is the dependent variable and adjustment is made for both admission assessment and length of time between assessments.

Data Management and Analysis will be completed using Stata version 10.0 software (Stata Corp, Texas).

#### Sample size

Recent economic modelling of patient education for prevention of falls indicates this intervention may be a cost-effective approach for the prevention of falls with reductions in the proportion of people who fall in the order of 20% and possibly as low as 10%[12]. Previous in-hospital investigations have demonstrated reductions in falls per 1000 patient days in the order of 30% using targeted, multifactorial interventions, [17] and amongst specific patient subgroups as high as 49% using patient education alone[27]. Given this background context, and that the education is being provided to a reasonably unselected patient cohort, we anticipated that this intervention could reduce the rate of falls per 1000 patient days by 30%.

We conducted 1000 bootstrap simulations of data previously published from this setting [17] and determined that our experiment would have 80% power to detect a reduction in the rate of falls of 30% in one of the intervention groups relative to the control group during the inpatient period. This assumes a sample size of n = 390 per group, that the control group has a rate of falls of 15.7 per 1000 patient days, use of a Cox semi-parametric proportional hazards regression (Anderson-Gill extension for recurrent events) analysis approach and a two-tailed alpha of 0.05. Given that previous research in this setting similar to the present trial has been completed with no drop-outs, [17] we will recruit only an additional 3% of patients per group culminating in a sample size of n = 402 per group (n = 1206 in total). This sample size will also have 80% power to detect a one-third reduction in the proportion of patients who are fallers assuming that 24% of those in the control group will become fallers during their hospitalisation. The follow-up cohort of n = 117 per group will have 80% power to detect a 33% reduction in the proportion who are fallers assuming that 58% of the control group become fallers during the 6 month follow-up [46].

#### Ethics Approval

Ethics approval was obtained from the Medical Research Ethics Committee at The University of Queensland (project number2007000148). The trial is registered with the Australian Clinical Trials registry: ACTRN12608000015347.

## Results

It is estimated that recruitment for the trial will be completed in April 2009. Data collection for the post discharge follow up cohort of 350 participants from SDH is anticipated to be completed by October 2009.

## Discussion

Patient behaviour is an integral contributing factor in many in-hospital falls.[7,24,25] In wards where patients are encouraged to regain their mobility and independence, a balance exists between encouraging patient activity and restricting activities to only those that a patient can perform safely. Staff commonly direct patients as to how much assistance they require, [17] however intrinsic patient factors such as cognitive impairment, inability to follow directions, confusion, and poor judgment may impede patient compliance with hospital staff recommendations. These factors have consistently been identified as significant risk factors for in-hospital fall s[47].

Falls risk reduction strategies such as alerting staff prior to mobilising and using equipment safely rely on patient adherence. Our education is based on a preventative health model of education [29] which proposes that patient adherence can be enhanced if patients have an awareness of the risks they face and furthermore have the knowledge and skills to perform strategies that could reduce their risk of falls. We argue that participants will be more likely to engage in safer behaviours when firstly they believe that the behaviour is likely to result in a beneficial outcome and secondly when they have sufficient self efficacy (confidence) to perform their strategies. Behaviour initiation may also be mediated by individual beliefs and approval of family and staff when participants undertake falls prevention strategies, thereby enhancing the desired behaviours. Finally our education aims to address barriers and misconceptions in a manner that enhances participants' comprehension and belief in the beneficial effect and achievability of falls prevention behaviours.

Previous studies have identified a consistent decline in functional status in older people post discharge [46,48] and a substantial increase in rates of falls in the first month after discharge.[11] Older patients may find it difficult to accurately assess their early mobility post injury or illness, when their functional status has not returned to pre-admission levels. Exploration of the link between participants' self-perceived risk of falls, knowledge of falls epidemiology, falls prevention strategies and motivation to undertake falls prevention strategies on discharge and participants' rates of falls and engagement in falls prevention activities after discharge will clarify the possible role of in-patient education in preventing falls in this population.

The strengths of this study include its size (n = 1206, the previous largest in this field with randomization of individual patients is n = 626), use of a randomised controlled trial approach, the use of multiple avenues for collating data on falls in hospitals, preparatory training of hospital staff to identify falls and use of assessors blinded to participant group allocation in the collation of outcome measures. The study has wide inclusion criteria for a hospital population of older people across two disparate geographical sites indicating that the findings of this research will be broadly applicable. The intervention employs a previously validated, empirically tested health education model as a framework for its development [29] This model for conceptualising health behaviours has previously been successfully used in a wide range of health areas, but to the researchers' knowledge this is the first time the HBM has been used in the area of falls prevention in hospital.

The study is not powered to show the effect of falls prevention education on falls injuries in hospital and after discharge. It has not been possible to blind ward staff to the intervention and it is possible that ward staff, after exposure to the intervention, have adapted their behaviour to study participants or to falls reporting. It is also possible that some participants discuss their strategies or treatment with participants allocated to the control group resulting in a dilution effect. Participants are drawn from a high risk population and continued participation may be affected by acute illness such that a participant becomes too medically unwell to receive the full intervention or is transferred to another hospital for urgent medical care and cannot continue to be monitored or receive the intervention.

## Conclusion

Uncertainty about the most effective interventions to reduce the high individual and societal cost of falls provides a compelling rationale for further large, well designed randomised trials to determine the most effective clinical interventions to reduce falls and falls injuries in older people, both in hospital and after discharge.[49] This is the first randomised controlled trial to examine the effect of patient education alone as an intervention to prevent falls in hospital and falls at home after discharge compared to providing usual care. The results of this study will provide robust recommendations for clinicians and researchers about the role of patient education in this population. This intervention is easily translated from the research to the clinical setting and could be duplicated across a very broad range of hospital environments.

## Competing interests

The authors declare that they have no competing interests.

## Authors' contributions

THa was principally responsible for the project conception and design of multimedia. AMH is responsible for project organisation, intervention provision and data collection at the SKHS site and SM at the PAH site. AMH and THa were principally responsible for the drafting of the manuscript. SM, KH, THo, SB, DO, CB, KM contributed to project conception and design and critical revision of the manuscript.

## Pre-publication history

The pre-publication history for this paper can be accessed here:



## Supplementary Material

Additional file 1 **Pre-hospital Discharge Survey**. Data collection; measurement tool – survey. Click here for file

Additional file 2 **Post Hospital Discharge 6 month Survey**. Data collection: measurement tool – survey. Click here for file
