# Supplementary material for: Evaluation of the effect of patient education on rates of falls in older hospital patients: Description of a randomised controlled trial
Source: BMC Geriatr. 2009 Apr 24;9:14. doi: 10.1186/1471-2318-9-14 (PMC2688498; doi:10.1186/1471-2318-9-14)
Supplement: Additional file 1 — Pre-hospital Discharge Survey. Data collection; measurement tool – survey. [file 1471-2318-9-14-S1.pdf]

APPENDIX FILE 1

(SDH site)

Pre-Hospital Discharge Survey

1) I think that older people who go home from hospital are at risk of falling over in the first 6 months.

Strongly agree Agree Undecided Disagree Strongly disagree

2) I think that I will fall over at some point in the first 6 months after I return home.

Strongly agree Agree Undecided Disagree Strongly disagree

3) I think that if a person falls over at home they are likely to get a mild injury (such as a skin cut or a bruise)

Strongly agree Agree Undecided Disagree Strongly disagree

4) I think if I were to fall over I would be likely to get a mild injury (such as a skin cut or a bruise)

Strongly agree Agree Undecided Disagree Strongly disagree

5) I think that if an older person falls over at home they are likely to get a serious injury (such as a sprain, bumped head or broken bone)

Strongly agree Agree Undecided Disagree Strongly disagree

6) I think that if I were to fall over in the first 6 months after going home from hospital, I would be likely to get a serious injury

Strongly agree Agree Undecided Disagree Strongly disagree

If you were to fall over while at home:

7) When would it most likely happen?

**Morning**<br><br>**6 – 10 am**	**Middle of day**<br><br>**10 am – 2 pm**	**Afternoon**<br><br>**2 – 6 pm**	**Evening**<br><br>**6 – 10 pm**	**Night time**<br><br>**10 pm – 2 am**	**Early morning**<br><br>**2 – 6 am**

8) Where would it most likely happen?

bedroom / bathroom or toilet / kitchen / living area / outdoor area around my home/ outside in community

9) Please state at least one strategy that you think might be able to reduce your risk of falls in the first 6 months after you go home from hospital

Open response. (more than one answer if possible)

10) I am confident that I could.............................(insert the most important thing mentioned) to prevent myself from falling when I went home from hospital

Strongly agree Agree Undecided Disagree Strongly disagree

11) Something that might make it difficult for me to do the above strategy is

Open response.

12) I am very motivated to lower my risk of falls at home in the first 6 months after hospitalisation by using these strategies (referring to the "most effective" strategy that the participant has just identified).

Strongly agree Agree Undecided Disagree Strongly disagree
