# Supplementary material for: Evaluation of the effect of patient education on rates of falls in older hospital patients: Description of a randomised controlled trial
Source: BMC Geriatr. 2009 Apr 24;9:14. doi: 10.1186/1471-2318-9-14 (PMC2688498; doi:10.1186/1471-2318-9-14)
Supplement: Additional file 2 — Post Hospital Discharge 6 month Survey. Data collection: measurement tool – survey. [file 1471-2318-9-14-S2.pdf]

**APPENDIX FILE 2**

(SDH site)

**Final 6month Post- Discharge Survey**

**1)** Tell me how much you agree or disagree with the following statement: "I think that I will fall over during the next 6 months." You can answer;

**Strongly agree Agree Undecided Disagree Strongly Disagree**

**2)** If you were to fall over at home in the future:

When do you think it would most likely happen?

| **Morning**<br><br>**6 – 10 am** | **Middle of day**<br><br>**10 am – 2 pm** | **Afternoon**<br><br>**2 – 6 pm** | **Evening**<br><br>**6 – 10 pm** | **Night time**<br><br>**10 pm – 2 am** | **Early morning**<br><br>**2 – 6 am** |
|---|---|---|---|---|---|

**3)** Where do you think it would most likely happen?

**bedroom / bathroom or toilet / kitchen / living area / outdoor area around my home/ outside in community / other…. (Record verbatim)**

**4)** Please tell me some things that you think other people like you could do to reduce their risk of falling over at home? (after first answer, follow with prompt - Is there anything else you think they could do? If people say – "just be careful", ask them to describe what this means to them or to give you an example)

**Open response. Write out a list of the things they say. If they give the "be careful" response, write this down and then write in brackets what this means to them or the examples they give)**

**5)** Which of these do you think would reduce their risk of falling over the most?

**Open response**

**6)** Tell me how much you agree or disagree with the following statement: "I think that if I did (*response to question 5*) I would reduce my own risk of falling." You can answer;

**Strongly agree Agree Undecided Disagree Strongly Disagree**

**7)** Tell me how much you agree or disagree with the following statement: "I am confident that I am able to (*response to question 5*)." You can answer;

**Strongly agree Agree Undecided Disagree Strongly Disagree**

**8)** Have you done this (*response to question 5*) yourself since you left the hospital 6 months ago?

☐ **I have done this regularly since leaving hospital** (go to qu. 10)

☐ **I did not start trying to do this straight away, but am doing it regularly now** (go to qu. 10)

☐ **I tried to do this earlier but am not doing it regularly at the moment** (go to qu. 9)

☐ **I have not tried to do this yet but I am planning to do so** (go to qu. 9)

☐ **I have not tried to do this and I do not plan to do so** (go to qu. 9)

**9)** Why do you think you are not doing (*response to question 5*) regularly at the moment? Is there anything else making it difficult for you to do this?

**Open response – try to write down answers verbatim as possible as these will later form quotes**

**10)** Participating in exercises aimed at improving balance and strength is recognised as a way to reduce the risk of falling. Can you remember being told that you should do exercise to improve your balance and strength by anyone either while you were in hospital or after you left.

**In hospital After discharge**

| | | |
|---|---|---|
| **Doctor / GP** | ☐ | ☐ |
| **Physio** | ☐ | ☐ |
| **Nurse** | ☐ | ☐ |
| **Other health professional** | ☐ | ☐ |
| **Family or friend** | ☐ | ☐ |

**11**) Tell me how much you agree or disagree with the following statement: "I think that if participated in exercises aimed at improving balance and strength I would reduce my own risk of falling." You can answer;

**Strongly agree Agree Undecided Disagree Strongly Disagree**

**12**) Tell me how much you agree or disagree with the following statement: "I am confident that I am able to perform exercises aimed at improving my balance and strength." You can answer;

**Strongly agree Agree Undecided Disagree Strongly Disagree**

**13)** Have you performed exercises aimed at improving my balance and strength since you left the hospital 6 months ago?

☐ **I have done this regularly since leaving hospital** (go to qu. 16)

☐ **I did not start trying to do this straight away, but am doing it regularly now** (go to qu. 16)

☐ **I tried to do this earlier but am not doing it regularly at the moment** (go to qu. 15)

☐ **I have not tried to do this yet but I am planning to do so** (go to qu. 15)

☐ **I have not tried to do this and I do not plan to do so** (go to qu. 15)

**14)** Why do you think you are not performing exercises aimed at improving your balance and strength regularly at the moment? Is there anything else making it difficult for you to do this?

**Open response, record verbatim – then go to qu.17.**

**15)** Please describe how you have been doing these exercises

**Open response, but try to classify under the following.**

☐ **Group based exercise program run by a health professional (eg. Physio, exercise physiologist)**

☐ **Group based exercise program run by a non-health professional (eg. Peer led)**

☐ **Being seen at home by health professional who provides exercises**

☐ **Doing an independent home exercise program prescribed by a health professional**

☐ **Doing exercises thought up by the patient / non-health professional (Eg. Family)**

**Other …. Record.**

**16)** I had a visit by the therapist from the hospital who arranged modifications to my home

**Yes** **No** **Unsure** **Had mods prior to discharge**

**17)** If Yes – can you tell me what they wanted to change?

**Open response**

**18)** Were all of these things changed? If no, why not?

**Open response**

**19)** How long has it been since you last had your eyes examined?

☐ **<6 months (ie. Since hospital discharge)**

☐ **6-12 months**

☐ **1 year – 5 years**

☐ **> 5 years**

**20)** Do you currently have a cataract or cataracts?

**Yes / No**

**21**) Have you previously had surgery to remove a cataract?

**Yes / No**

**22**) Have you bought new walking shoes in the past 6 months

**Yes / No**

**23)** Taking sleeping medication has been shown to increase risk of falls. Can you tell me if you were taking sleeping medication when you left hospital? If so, have you reduced or stopped taking sleeping medication in the past 6 months (since you left hospital)?

**Yes / No / Was not taking sleeping medication in the first place**

**24)** Taking lots of medication has been shown to increase risk of falls. Can you tell me if you were taking more than 4 medications regularly when you left hospital? Have you talked to your GP or pharmacist about reducing the number of medications you are taking in the past 6 months (since you left hospital)

**Yes / No / Was not taking more than 4 medications in the first place**

**25)** People who have difficulty with showering and getting dressed have been found to have a higher risk of falls. Do you think that you are having difficulty with this at the moment? Are you currently receiving help with showering or getting dressed?

**Yes / Yes but not now / No - but I am having trouble / No – but I don't think I am having trouble**

**26)** Since leaving hospital 6 months ago, have you had to go to hospital again?

**Yes / No**

If yes –

Number of days with overnight stay ………………….. & diagnosis (or best description of diagnosis possible) …………………………………..

Number of presentations without overnight stay ………………. & diagnosis ………………………..

**27)** Do you have any comment on prevention of falls in the home for older people after they come home from hospital?

**Record verbatim.**

___________________________________________________________________________
